# Standardized Near-Peer Clinical Skills Training for Seven Student-Run Clinics: Assessing Impact on Student Confidence

**DOI:** 10.7759/cureus.114881

**Published:** 2026-08-20

**Authors:** Jared Boetes, Parker Kronen, Kyle Polen, Cindy W Christian, Horace M DeLisser

**Affiliations:** 1 Academic Programs Office, University of Pennsylvania Perelman School of Medicine, Philadelphia, USA

**Keywords:** access to healthcare, community engagement, community service, medical student run clinics, service learning

## Abstract

Background

First-year medical students volunteering in student-run clinics (SRCs) often perform clinical skills before they are formally introduced in the curriculum. At some institutions, students are trained on an ad hoc basis by individual clinics without centralized oversight or quality control. Near-peer teaching models have shown promise in practical skills training and may benefit pre-clerkship learners prior to volunteering at SRCs. The objective of this study was to evaluate whether a standardized and centrally coordinated near-peer clinical skills curriculum (including modules on Vital Signs, History Taking and Patient Presentations, and Physical Exam) improved medical students’ self-reported confidence for SRC work across seven different clinics.

Methods

Three standardized training modules (Vital Signs, History Taking and Patient Presentations, and Physical Exam) were developed and facilitated by near-peer educators. Each module consisted of didactic instruction followed by hands-on practice and assessment, with close guidance from near-peer trainers. First-year students completed surveys pre-training, post-training, and two months after training ("post-clinic”) that assessed self-reported confidence and perceived effectiveness of the training model using 5-point Likert scales.

Results

There were highly significant confidence improvements across all three training modules (p<0.01). Vital signs confidence increased from 2.69 pre-training to 4.60 post-training (paired n=65) and held at 4.48 post-clinic (paired n=25). History taking and patient presentation confidence increased from 3.03 pre-training to 4.44 post-training (paired n=59) and held at 4.17 post-clinic (paired n=18). Physical exam confidence increased from 2.08 pre-training to 4.46 post-training (paired n=26); post-clinic response rate was insufficient for meaningful analysis. Across the three training modules, all students agreed that the near-peer teaching model was effective for learning how to perform the respective clinical skills, with 92.3% of students in the Vital Signs training (n=65), 86.4% in the History Taking and Patient Presentations training (n=59), and 80.8% in the Physical Exam training (n=26) strongly agreeing.

Conclusions

Standardized clinical skills training led by near-peer educators significantly improves confidence across three core domains for first-year medical students volunteering at multiple SRCs. This scalable model empowers near-peer educators to create accessible learning environments that supplement the curriculum. Future work will include objective clinical performance assessments to validate whether self-reported confidence translates to enhanced clinical skill competence and improved patient outcomes.

## Introduction

Student-run clinics (SRCs) are organizations managed by medical and other health professions students that aim to improve healthcare access for traditionally underserved communities [1]. As of 2025, there are an estimated 500 SRCs across 42 U.S. states and 11 countries, and the number continues to grow [2]. At MD and DO programs, SRCs provide early-career medical students with valuable opportunities to practice core skills prior to their clinical clerkships [1]. However, some students participate in SRCs so early in their education that they perform these clinical skills on SRC patients before they are formally taught in the curriculum. At some institutions, students are trained on an ad hoc basis by their respective SRCs to address existing knowledge gaps, but these trainings are often done without centralized oversight or quality control. Therefore, patients receiving care from SRCs often interact with students who have no or very limited clinical skills training, and the degree of supervision by residents and attending physicians can vary clinic to clinic [3].

Given that SRC patient populations tend to be underserved, under-resourced, and ethnically and racially diverse, the variability in students’ clinical training can be ethically problematic, raising questions about healthcare equity and the impact on patient welfare [4]. Without sufficient training, students volunteering in SRCs can feel insecure about their ability to provide high-quality patient care, thus resulting in moral distress and a suboptimal experience for learners and patients alike [5]. Among the various teaching models that have been assessed for their efficacy in medical student clinical training, peer-assisted learning has been shown to be significantly effective for practical skills acquisition [6,7]. While peer-assisted learning and SRC training models have been described previously, most published interventions focus on individual clinics or specific educational activities [8-10]. There remains limited evidence evaluating standardized, centrally coordinated training curricula that prepare pre-clinical students for participation across multiple SRCs. We therefore studied the impact of a near-peer designed and led clinical skills training program which was tailored to the specific responsibilities of early, pre-clinical medical students volunteering across multiple SRCs. The objective of this study was to evaluate whether a standardized and centrally coordinated near-peer clinical skills curriculum (including modules on Vital Signs, History Taking and Patient Presentations, and Physical Exam) improved medical students’ self-reported confidence for SRC work across seven different clinics.

## Materials and methods

Overview of the study

This was a single-institution, single-arm pre-post quality improvement study without a control group. The primary objective of this study was to evaluate whether a standardized and centrally coordinated near-peer clinical skills curriculum improved medical students’ self-reported confidence for work across seven SRCs. The secondary objectives were to assess the perceived effectiveness of the near-peer training model and the perceived support from the near-peer educators. The study was conducted at the Perelman School of Medicine at the University of Pennsylvania, where first-year students were offered volunteer opportunities across seven SRCs within the first two months of their medical education, prior to formally being taught clinical skills in the curriculum (Table 1).

**Table 1:** Student-run clinics that participated in the study at the University of Pennsylvania Perelman School of Medicine.

Clinic Name	Population Served	Care Provided
CUT Hypertension	Local Philadelphia residents	Blood pressure screening and information about hypertension
Homeless Outreach Project	People experiencing homelessness	Primary care services, wound care, and ultrasound imaging
Q-munity Wellness Clinic	LGBTQ+ individuals in Philadelphia	LGBTQ+ affirming psychiatric evaluations, medication management, and psychotherapy
Refugee Health Clinic	Newly arrived refugees from Asia, Africa, and the Middle East	Primary care services, immunizations, and infectious and chronic disease screening
United Community Clinic	Underinsured/uninsured local residents; African and Caribbean immigrants and refugees	Primary care services, immunizations, and infectious and chronic disease screening
Unity Health Clinic	Southeast Asian immigrants	Primary care services, immunizations, and infectious and chronic disease screening
University City Hospitality Coalition	People experiencing homelessness; underinsured/uninsured local residents	Primary care services, infectious and chronic disease screening, and specialty care

At these SRCs, patient care is supervised by licensed physicians, and several unique populations are served, including local West Philadelphia residents, African and Caribbean immigrants, Southeast Asian immigrants, refugees, LGBTQ+ individuals, and people experiencing homelessness. This study was approved by the University of Pennsylvania Institutional Review Board (IRB) as a quality improvement initiative that did not meet the definition of human subjects research and was therefore exempted from further IRB review. Students were encouraged to participate in all the training modules but could choose to participate in any number of the modules. All sample sizes represent totals of unique participants. Demographic data (race, ethnicity, gender, age, etc.) were not collected as these data were not pertinent or necessary for the training that was provided.

Description of the training program

A team of three third-year medical students created training modules for the following skills: Vital Signs, History Taking and Patient Presentations, and Physical Exam. The content of each module was reviewed and approved by a physician who serves as the medical school’s Assistant Dean of Community Engagement, overseeing all SRCs. SRC volunteers participated in at least one of the three trainings (Vital Signs, History Taking and Patient Presentations, and Physical Exam) depending on the student’s responsibilities at their respective SRC(s). Each training session consisted of didactics (with associated informational slides) and hands-on practice. Didactics were delivered by medical students who were one or more years senior to the trainees, and there was a trainee-to-trainer ratio of 2:1 during hands-on practice. For each training session, the trainers met 30 minutes prior to review content, learning objectives, and criteria for competency checks. There were no residents, attending physicians, or other faculty present at the training sessions. The training environment was designed to minimize stress so that trainees would feel empowered to practice repeatedly and ask questions to optimize their performance.

Vital Signs training session

Measurements of heart rate, respiratory rate, and blood pressure were the focus of the Vital Signs training. Each of the aforementioned skills was taught and practiced before moving on to the next. Didactics focused on proper technique, best practices, and normal ranges for each measurement. Trainees used their personal stethoscopes, and aneroid sphygmomanometers were provided. Trainees were paired up at random and practiced measuring each other’s vital signs manually with guidance from a near-peer educator. To pass the training, trainees had to satisfactorily measure heart rate, respiratory rate, and blood pressure in succession on three different near-peer trainers. The near-peer trainers measured their heart rate and blood pressure in advance; therefore, satisfactory measurements were determined to be within 10 beats per minute and 10 mmHg of the trainers’ known heart rate and blood pressure, respectively, to account for the trainers’ natural fluctuations.

History Taking and Patient Presentations training session

Trainees first participated in didactics, which focused on key points for history taking (introducing oneself to the patient, obtaining a chief complaint and history of present illness, and concluding the interview), as well as formulating a one-liner to begin a patient presentation. Trainees were then paired at random and asked to interview each other. Trainees alternated between playing the role of interviewer in one encounter, then patient in another. When playing the role of patient, trainees were able to reference a document that outlined their full history and review of systems. Upon completing the interview, patients gave feedback to the interviewers, and each interviewer briefly presented their patient. Near-peer educators supervised the interviews, provided guidance as needed, and played the role of attending physician when an interviewer presented a patient. To pass the training, trainees had to satisfactorily complete two interviews (once as an interviewer, once as a patient) and present a patient. A satisfactory patient presentation contained at least 80% of the relevant information gathered during the interview, as determined by the near-peer educators.

Physical Exam training session

This training focused on heart auscultation, lung auscultation, and abdomen auscultation and palpation. Similar to the Vital Signs training, each of the aforementioned skills was taught and practiced before moving on to the next, and trainees used their personal stethoscopes. Didactics focused on anatomic landmarks, proper technique, best practices, and normal versus pathological findings. Due to the more intimate nature of some of the physical exam maneuvers, near-peer educators served as patients on whom the trainees could practice. To pass the training, trainees had to satisfactorily perform all four physical exam skills in succession on three different near-peer trainers. For a physical exam to be deemed satisfactory, the student was required to introduce themselves, guide the near-peer trainer through each maneuver that was about to be performed, and use appropriate technique for all maneuvers.

Evaluation of training program

For each of the training sessions, trainees were asked to complete three surveys: one immediately before the training (“pre-training”), one immediately after the training (“post-training”), and one two months after the training once the skills had been performed on a real SRC patient (“post-clinic”). The surveys used 5-point Likert scales to assess the perceived effectiveness of the training model as well as the trainees’ self-reported confidence in performing the clinical skills that were taught (see Appendices). Participants’ responses were matched across the three surveys, and differences between survey time points were analyzed using Wilcoxon signed-rank tests in R (R Foundation for Statistical Computing, Vienna, Austria).

## Results

Vital Signs training

**Figure 1:**
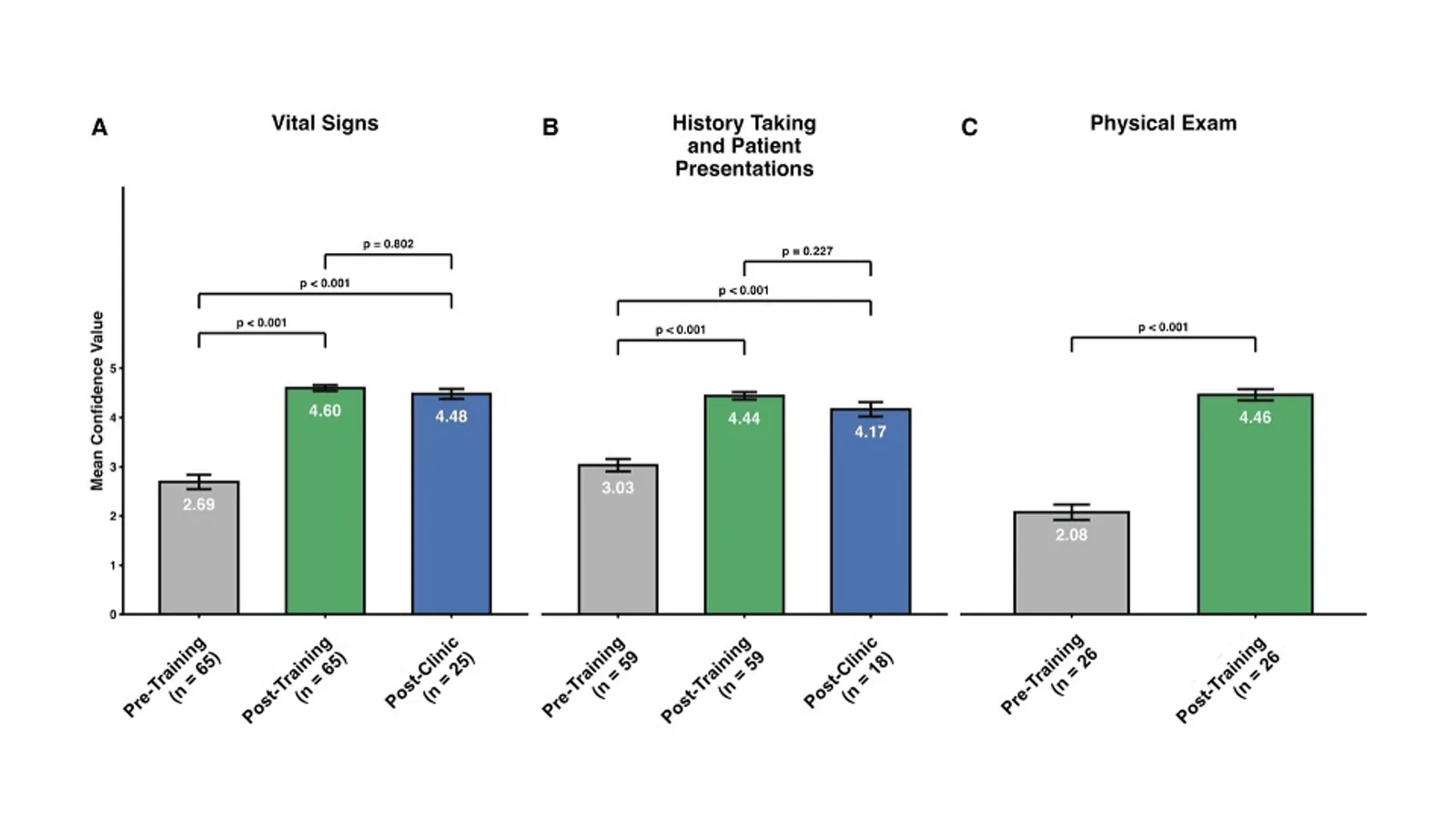
Post-training and post-clinic confidence. Post-training and post-clinic increases in mean confidence were achieved across all three training modules. P-values were calculated using Wilcoxon signed-rank tests for participants with matched responses at the two time points being compared.

A total of 65 students participated in Vital Signs training. Thirty-four students (52.3%) had prior experience taking vital signs, with the three most common experiences being physician shadowing (13 students), emergency medical technician (10 students), and medical assistant (10 students). All participants passed the training. Response rates for the pre-training, post-training, and post-clinic surveys were 100%, 100%, and 38.5%, respectively. Mean confidence increased from 2.69 (SD 1.18; median 3, IQR 2-4) pre-training to 4.60 (SD 0.49; median 5, IQR 4-5) post-training (Wilcoxon signed-rank test, paired n=65, p<0.001). Among students who performed vital signs on an SRC patient and responded to the post-clinic survey, mean confidence remained significantly elevated at 4.48 (SD 0.51; median 4, IQR 4-5) post-clinic (Wilcoxon signed-rank test, paired n=25, p<0.001) (Figure 1A). The difference between post-training and post-clinic mean confidence was not statistically significant (Wilcoxon signed-rank test, paired n=25, p=0.802).

History Taking and Patient Presentations training

A total of 59 students participated in History Taking and Patient Presentations training. Forty students (67.8%) had prior experience taking and presenting a patient history, with the three most common experiences being physician shadowing (19 students), medical assistant (13 students), and emergency medical technician (12 students). All participants passed the training. Response rates for the pre-training, post-training, and post-clinic surveys were 100%, 100%, and 30.5%, respectively. Mean confidence increased from 3.03 (SD 0.98; median 3, IQR 2-4) pre-training to 4.44 (SD 0.60; median 4, IQR 4-5) post-training (Wilcoxon signed-rank test, paired n=59, p<0.001). Among students who took a history from a SRC patient, presented the patient, and responded to the post-clinic survey, mean confidence remained significantly elevated at 4.17 (SD 0.62; median 4, IQR 4-4.75) post-clinic (Wilcoxon signed-rank test, paired n=18, p<0.01) (Figure 1B). The difference between post-training and post-clinic mean confidence was not statistically significant (Wilcoxon signed-rank test, paired n=18, p=0.227).

Physical Exam training

A total of 26 students participated in Physical Exam training. Nine students (34.6%) had prior experience performing a physical exam, with the three most common experiences being physician shadowing (4 students), emergency medical technician (4 students), and medical assistant (2 students). All participants passed the training. Response rates for the pre-training, post-training, and post-clinic surveys were 100%, 100%, and 7.7%, respectively. Mean confidence increased from 2.08 (SD 0.80; median 2, IQR 1.25-3) pre-training to 4.46 (SD 0.58; median 4.5, IQR 4-5) post-training (Wilcoxon signed-rank test, paired n=26, p<0.001) (Figure 1C). The post-clinic survey sample size was considered insufficient for meaningful analysis.

Near-peer teaching model

**Figure 2:**
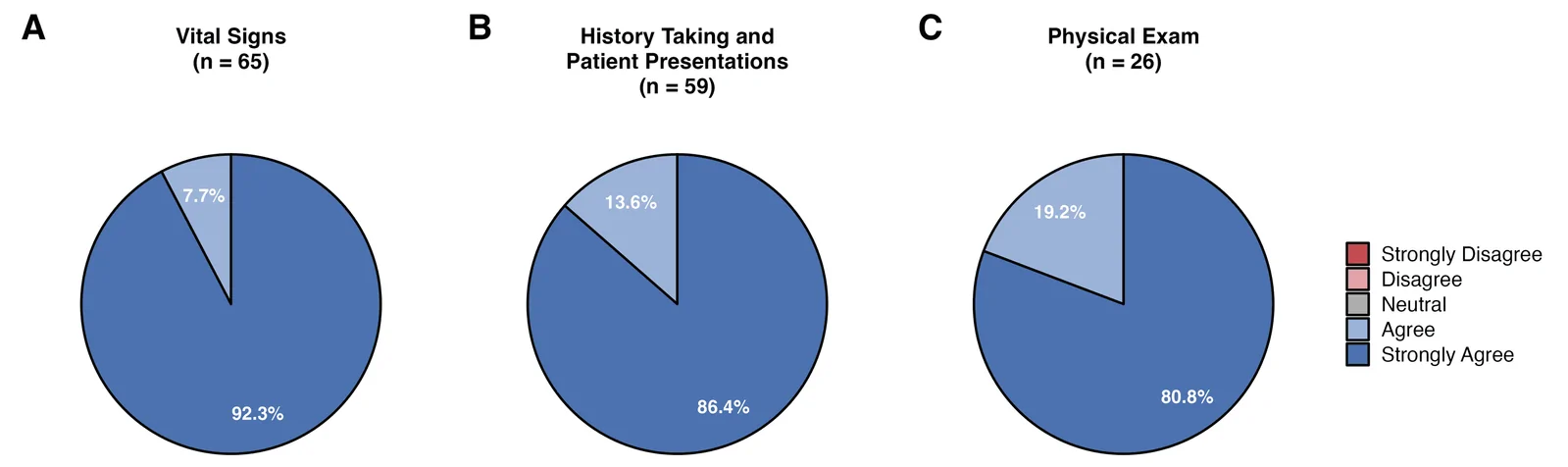
Perceived effectiveness of the near-peer teaching model across all three training modules.

Across the three training modules, all students agreed that the near-peer teaching model was effective for learning how to perform the respective clinical skills, with 92.3% of students in the Vital Signs training (n=65), 86.4% in the History Taking and Patient Presentations training (n=59), and 80.8% in the Physical Exam training (n=26) strongly agreeing (Figure 2).

**Figure 3:**
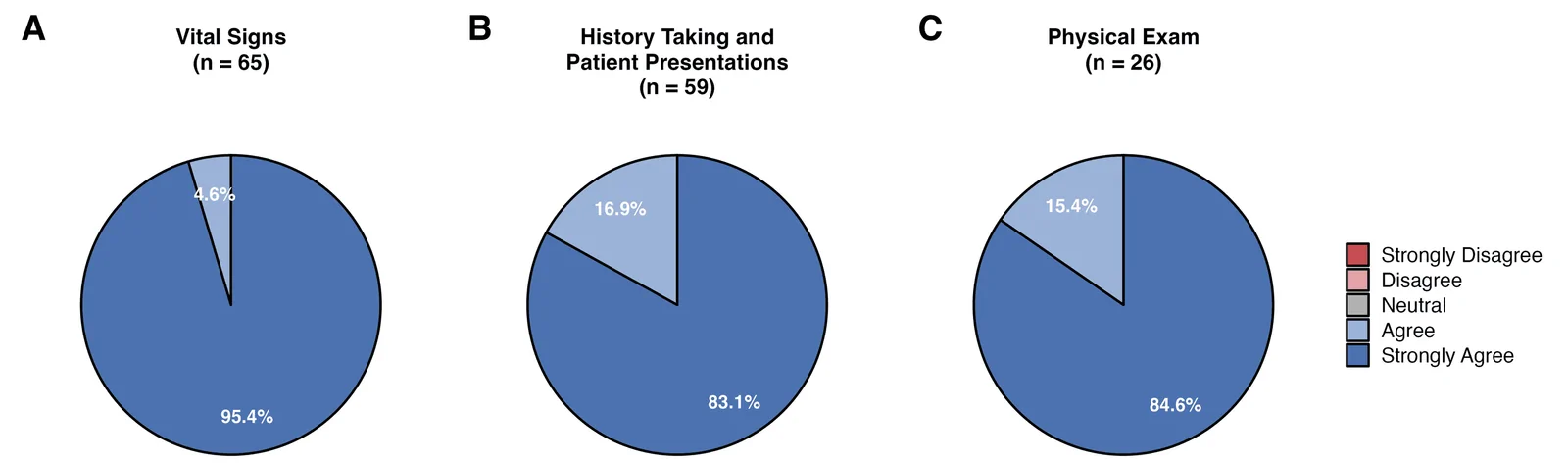
Perceived support from near-peer educators across all three training modules.

Additionally, all students agreed that they felt supported when working with near-peer educators, with 95.4% of students in the Vital Signs training (n=65), 83.1% in the History Taking and Patient Presentations training (n=59), and 84.6% in the Physical Exam training (n=26) strongly agreeing (Figure 3).

## Discussion

We implemented a standardized and centrally coordinated training program in which near-peer educators taught vital signs, history taking and patient presentations, and physical exam skills to first-year medical students to prepare them for volunteer work at seven SRCs. Across all three modules, self-reported confidence increased significantly from pre- to post-training (p<0.01), and the confidence improvements were sustained two months later among students who subsequently performed the skills on patients in the SRC setting. Trainees also strongly endorsed the near-peer teaching model.

Peer-assisted learning is well-established in undergraduate medical education, with prior literature demonstrating improvements greater than traditional teaching methods, particularly for practical skills [6,7,11]. Moreover, there is strong theoretical support for the benefits of near-peer teaching compared to faculty teaching, as the shared experiences between learners and near-peer educators result in social and cognitive congruence. Near peers are uniquely capable of mentoring and empathizing with learners because they are more familiar with the expectations and pitfalls that may be encountered during the earlier stages of training [12,13]. Separately, SRCs are recognized as valuable environments for preclinical skills development and have been shown to improve self-reported confidence and clerkship preparedness among student volunteers [10,14-16]. However, the intersection of peer-assisted learning and SRCs in the literature is underdeveloped: most SRCs train volunteers on an ad hoc basis, and there are few published models demonstrating how to standardize early clinical skills training across multiple clinics for students who will see patients before formal curricular instruction [17]. Our model addresses this gap by applying a centralized, multi-skill peer-assisted learning curriculum upstream of SRC participation rather than relying on individual clinics to design their own training. Furthermore, the structure of brief didactics, hands-on practice with a 2:1 trainee-to-trainer ratio, and competency checks by multiple near-peer trainers is low-resource and reproducible.

This study has several limitations. First, the outcomes are based on self-reported confidence rather than objective performance, and increased confidence does not imply competence or better patient care. Additionally, because outcomes were based on self-reported survey responses, findings may have been affected by response and social desirability biases. Selection bias may have also occurred if students who elected to participate differed systematically from those who did not. Students’ prior experience performing certain skills may have confounded the results as well. Post-clinic response rates were lower than post-training response rates across all modules, and the post-clinic survey data were insufficient for meaningful analysis in the Physical Exam cohort. This was largely the result of some students not having the opportunity to volunteer at their respective SRCs prior to the post-clinic surveys being distributed. Because the study was conducted at a single institution with a specific SRC structure and curricular timing, the findings may be less generalizable to institutions with different SRC structures, supervision models, or timing of clinical skills curricula. Finally, we did not collect data on patient outcomes, and the absence of a comparison group precludes any inference about training effects relative to no training or to faculty-led alternatives.

Future work should focus on clinical performance and patients’ experiences. Preceptor evaluations of trainees during SRC encounters would provide another assessment of whether confidence translates to competent execution of clinical skills. Patient-reported outcomes, including measures of communication, comfort, and perceived quality of care, would help determine whether the benefits of standardized training are detectable from the patient's perspective. Longer-term follow-up into the clerkship year could also assess whether early near-peer-led training impacts skill development once students enter formal clinical training.

## Conclusions

A standardized and centrally coordinated near-peer clinical skills curriculum improved first-year medical students' self-reported confidence across three core clinical skill domains, and the confidence improvements were largely sustained after students performed these skills in several SRC settings. The model is low-resource, scalable, and well-received by trainees, and it may offer a practical way for medical schools with multiple SRCs to provide consistent early clinical skills preparation for students who volunteer before formal curricular instruction. 

## Appendices

Study surveys

**Table 2:** Study surveys. For each of the three training sessions, trainees were asked to complete three surveys: one immediately before the training (Pre-Training), one immediately after the training (Post-Training), and one two months after the training, once the skills had been performed on a patient in a student-led clinic (Post-Clinic).

Pre-Training Survey	Question	Options for Response
	1. Have you had previous experience taking vital signs on a patient manually? If yes, please indicate the role in which you performed this skill. Otherwise, select “No prior experience.”	a. No prior experience; b. Nurse; c. Emergency Medical Technician (EMT); d. Medical Assistant; e. Certified Nursing Assistant (CNA); f. Patient Care Technician (PCT); g. Home Health Aide; h. Physician shadowing; i. Other
	2. If you selected “Other,” please write the role in which you practiced this skill.	(Free text)
	3. I feel confident taking vital signs on a patient manually.	a. Strongly disagree; b. Disagree; c. Neutral; d. Agree; e. Strongly agree
Post-Training Survey	Question	Options for Response
	1. Upon completing the training, I feel confident in my ability to take vital signs on a patient manually	a. Strongly disagree; b. Disagree; c. Neutral; d. Agree; e. Strongly agree
	2. I felt supported working with upperclassmen medical students during the training.	a. Strongly disagree; b. Disagree; c. Neutral; d. Agree; e. Strongly agree
	3. Working with upperclassmen medical students was an effective way to learn how to take vital signs manually.	a. Strongly disagree; b. Disagree; c. Neutral; d. Agree; e. Strongly agree
Post-Clinic Survey	Question	Options for Response
	1. Since completing the training, have you taken vital signs on a patient manually in a clinical setting (excluding Introduction to Clinical Medicine, standardized patient encounters, etc.)?	a. Yes; b. No
	2. Upon working with patients, I felt confident in my ability to take vital signs manually because of the training.	a. Strongly disagree; b. Disagree; c. Neutral; d. Agree; e. Strongly agree
